# Early adaptation to eolian sand dunes by basal amniotes is documented in two Pennsylvanian Grand Canyon trackways

**DOI:** 10.1371/journal.pone.0237636

**Published:** 2020-08-19

**Authors:** Stephen M. Rowland, Mario V. Caputo, Zachary A. Jensen

**Affiliations:** 1 Department of Geoscience, University of Nevada Las Vegas, Las Vegas, Nevada, United States of America; 2 Las Vegas Natural History Museum, Las Vegas, Nevada, United States of America; 3 Pacific Section SEPM (Society for Sedimentary Geology), Tujunga, California, United States of America; 4 Department of Physical Sciences, College of Southern Nevada, Las Vegas, Nevada, United States of America; University of Wisconsin Madison, UNITED STATES

## Abstract

We report the discovery of two very early, basal-amniote fossil trackways on the same bedding plane in eolian sandstone of the Pennsylvanian Manakacha Formation in Grand Canyon, Arizona. Trackway 1, which is *Chelichnus*-like, we interpret to be a shallow undertrackway. It displays a distinctive, sideways-drifting, footprint pattern not previously documented in a tetrapod trackway. We interpret this pattern to record the trackmaker employing a lateral-sequence gait while diagonally ascending a slope of about 20°, thereby reducing the steepness of the ascent. Trackway 2 consists only of aligned sets of claw marks. We interpret this trackway to be a deeper undertrackway, made some hours or days later, possibly by an animal that was conspecific with Trackmaker 1, while walking directly up the slope at a speed of approximately 0.1 m/sec. These trackways are the first tetrapod tracks reported from the Manakacha Formation and the oldest in the Grand Canyon region. The narrow width of both trackways indicates that both trackmakers had relatively small femoral abduction angles and correspondingly relatively erect postures. They represent the earliest known occurrence of dunefield-dwelling amniotes―either basal reptiles or basal synapsids―thereby extending the known utilization of the desert biome by amniotes, as well as the presence of the *Chelichnus* ichnofacies, by at least eight million years, into the Atokan/Moscovian Age of the Pennsylvanian Epoch. The depositional setting was a coastal-plain, eolian dunefield in which tidal or wadi flooding episodically interrupted eolian processes and buried the dunes in mud.

## Introduction

**Fig 1:**
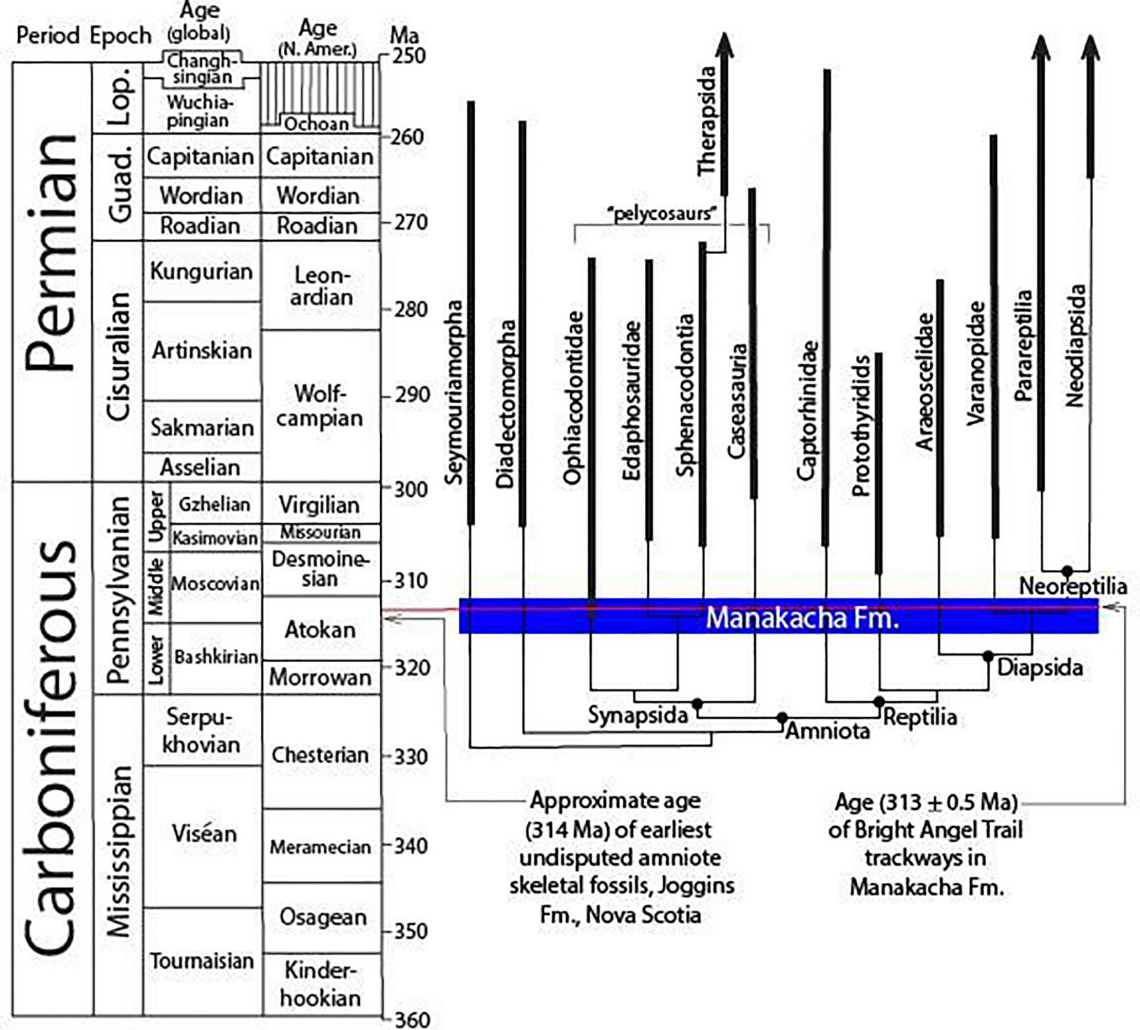
Stratocladogram of Carboniferous and Permian tetrapod phylogeny showing age of Manakacha Formation and Bright Angel Trail trackways. Stratocladogram is adapted from [4]. Time scale is from [12]. Guad., Guadalupian; Lop., Lopingian.

Amniotes evolved early in the Pennsylvanian or late in the Mississippian Epoch [1–4]. The earliest undisputed evidence of their presence―skeletal remains in the Joggins Formation of Nova Scotia―are dated at about 314 Ma, in the Moscovian/Atokan Stage [5–7] (Fig 1). It is widely accepted that basal amniotes rapidly radiated into several clades early in their history, and that the initial stages of this radiation are not well represented in the skeletal record [4,6,8]. Due in part to the problem of ghost lineages representing the early history of several clades of amniotes and diadectomorph amphibians (Fig 1), the study of fossil trackways has become a valuable supplement to skeletal data for teasing the early phylogenetic, paleoecologic, and biomechanical history of these groups out of the geologic record [7,9–11]. Until now, trackways of amniotes have not been reported from eolian environments prior to late in the Pennsylvanian.

The nurseries of amniote evolutionary innovation were Carboniferous tropical forests [3]. The freedom from having to lay their eggs in water permitted basal amniotes to inhabit terrestrial settings that were ecologically inaccessible to amphibians [2,3]. The trackway record indicates that, very early in amniote history, some taxa became adapted to seasonally dry, alluvial-plain settings [7,9,13], thus establishing an environmental beachhead from which their descendants were able to expand into even drier biomes, such as desert and coastal sand dunes.

As the supercontinent Pangaea began to assemble, beginning in the mid-Paleozoic Era, Europe and North America became sutured together into a continuous Euramerican landmass. In mid-Carboniferous time Euramerica was united with other continents to form Pangaea [14]. During the Pennsylvanian Epoch, this nascent supercontinent drifted slowly northward [15].

All known Pennsylvanian tetrapod trackways occur in either Euramerica [10,16] or Morocco [17,18]. These primarily consist of a mixture of temnospondyl amphibian and captorhinomorph reptile tracks, with the latter becoming more abundant in the Middle Pennsylvanian [10]. This trend continues into the lower portions of the Upper Pennsylvanian, but with the addition of characteristic Lower Permian ichnotaxa such as *Batrachichnus*, *Ichniotherium*, *Dromopus*, and *Dimetropus* [10].

Many Pennsylvanian tracksites are associated with coal beds [19], with the coal fields of Nova Scotia being an especially rich source of vertebrate footprints [20]. Non-coal-forming Pennsylvanian depositional environments have also yielded tetrapod trace fossils, including forested, poorly-drained coastal plains and well-drained alluvial plains [7,13,21]. However, conspicuously absent among these eastern North American, European, and Moroccan Pennsylvanian tetrapod tracksite localities are eolian lithofacies. The only well established occurrence of tetrapod tracks within eolian sandstone of possible Pennsylvanian age is in western North America, in the Pennsylvanian-Permian Weber Sandstone of northeastern Utah [22]. The age of the Weber Sandstone tracks is poorly constrained; they are probably post-Desmoinesian (post-upper Middle Pennsylvanian) [22].

Anther possible case of Pennsylvanian tetrapod tracks in eolian sandstone is in the Tensleep Sandstone of western Wyoming [23]. These tracks, which represent the only known occurrence of the ichnogenus *Steganoposaurus*, were originally interpreted as amphibian tracks [23], however Lockley and Hunt [24] reinterpreted them to be primitive reptile tracks. The stratigraphic position and the depositional environment of the Tensleep tracks have apparently not been studied in detail. The formation contains eolian deposits in its upper (Missourian and Virgilian) parts [25], but it is not clear from the published literature whether the *Steganoposaurus* trackway occurs within this eolian interval. The original authors [23] interpreted the single *Steganopoosaurus* trackway to record an animal emerging from a pond. This would not preclude an eolian setting, but neither is it typical of such an environment. If this trackway does indeed occur in eolian sediments, it is most probably Missourian or Virgilian (Upper Pennsylvanian) in age, which is roughly equivalent in age to the Weber Sandstone tracks.

Prior to the Pennsylvanian, eolian deposits were not abundant globally [26,27]. In North America specifically, within the Phanerozoic stratigraphic record, pre-Pennsylvanian eolian deposits are restricted to the Cambrian and Ordovician of the upper Mississippi Valley, and a Mississippian-age interval in western Wyoming [26]. However, beginning in the Pennsylvanian and continuing through the Jurassic, due to tectonic, paleogeographic, and sea-level factors, the eolian dunefield became a recurring depositional environment, especially in the region of Pangaea that is now southwestern North America [26,27]. This region lay in a favorable wind regime during the late Paleozoic and early Mesozoic, and tectonism associated with the assembly and subsequent fragmentation of Pangaea created an abundant supply of sand [28]. During the Pennsylvanian, eustatic sea level dropped due to glaciation in high southern latitudes [14,29]. Pennsylvanian marine strata in the southern Great Basin, west of Grand Canyon, record a gradual, long-term fall in relative sea level through the late Morrowan and Atokan ages, culminating in widespread exposure of portions of the craton near the end of the Atokan [30]. This exposed expansive, formerly marine areas to fluvial and eolian processes, resulting in the development of flood plains, wadis, and associated dunefield settings in the western interior of North America. As we discuss in greater detail later in this paper, the Manakacha Formation, exposed in Grand Canyon, is a product of this sequence of events.

Reptiles are archetypical vertebrate residents of dunefield habitats today, and one might hypothesize that their Pennsylvanian, basal-amniote ancestors would have quickly adapted to this setting. Eolianites are notoriously depauperate with respect to body fossils, but they are surprisingly rich in trace fossils; there is a prolific literature documenting the occurrence of vertebrate and arthropod trackways in eolianites [e.g., 24,31–34]. If amniotes were able to quickly adapt to eoian dunefields, there should be a record of their presence in the form of fossil trackways in mid-Pennsylvanian eolianites. But the earliest reported occurrence of amniote tracks in eolianite is quite late, relative to the occurrence of amniotes in other environments; it occurs in the Pennsylvanian-Permian Weber Sandstone, discussed above [22]. At a minimum, the Weber Sandstone tracks postdate the origin of amniotes by eight million years. Here we document the presence of amniote trackways in much older eolianite.

## Material and methods

In this study we follow the standard protocol recommended by Falkingham et al. [35] for documenting ichnological data. Field work was conducted under field permit GRCA-2017-SCI-0054 to SMR from the National Park Service.

**Fig 2:**
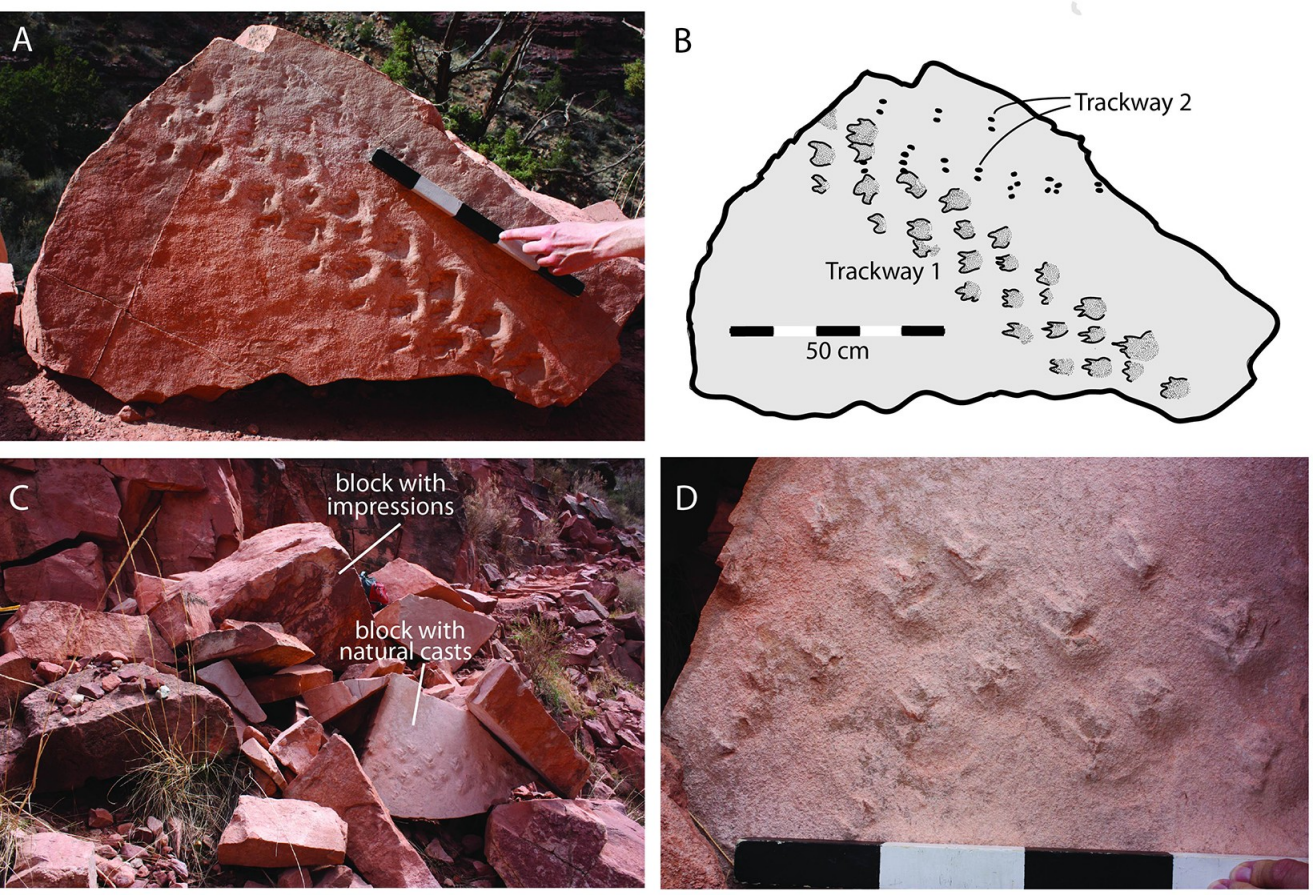
Trackway-bearing blocks. (*A*) Main trackway block adjacent to Bright Angel Trail, with tracks in concave epirelief (impressions). Scale is calibrated in decimeters. (*B*) Sketch of main trackway surface. Note occurrence of Trackway 2 (alignments of small black spots) above Trackway 1. (*C*) Jumble of rocks adjacent to Bright Angel Trail, including at least two rocks with amniote tracks. (*D*) Counterpart block with tracks in convex hyporelief (natural casts). Scale is calibrated in decimeters.

A recent rockfall from an exposed interval of the Manakacha Formation, adjacent to the heavily-traveled Bright Angel Trail in Grand Canyon National Park, produced a jumble of blocks of eolian sandstone (Fig 2). Two of the blocks contain corresponding part-and-counterpart, bedding-plane surfaces on which two vertebrate trackways occur: a very conspicuous trackway, here referred to as Trackway 1, consisting of plantigrade footprints with prominent claw marks (Fig 2A and 2B), and a less conspicuous trackway, here referred to as Trackway 2, consisting of two rows of paired, oval pits (Fig 2B). The two blocks evidently split apart along the trackway-bearing bedding plane when the parent rock fell from the cliff face.

**Fig 3:**
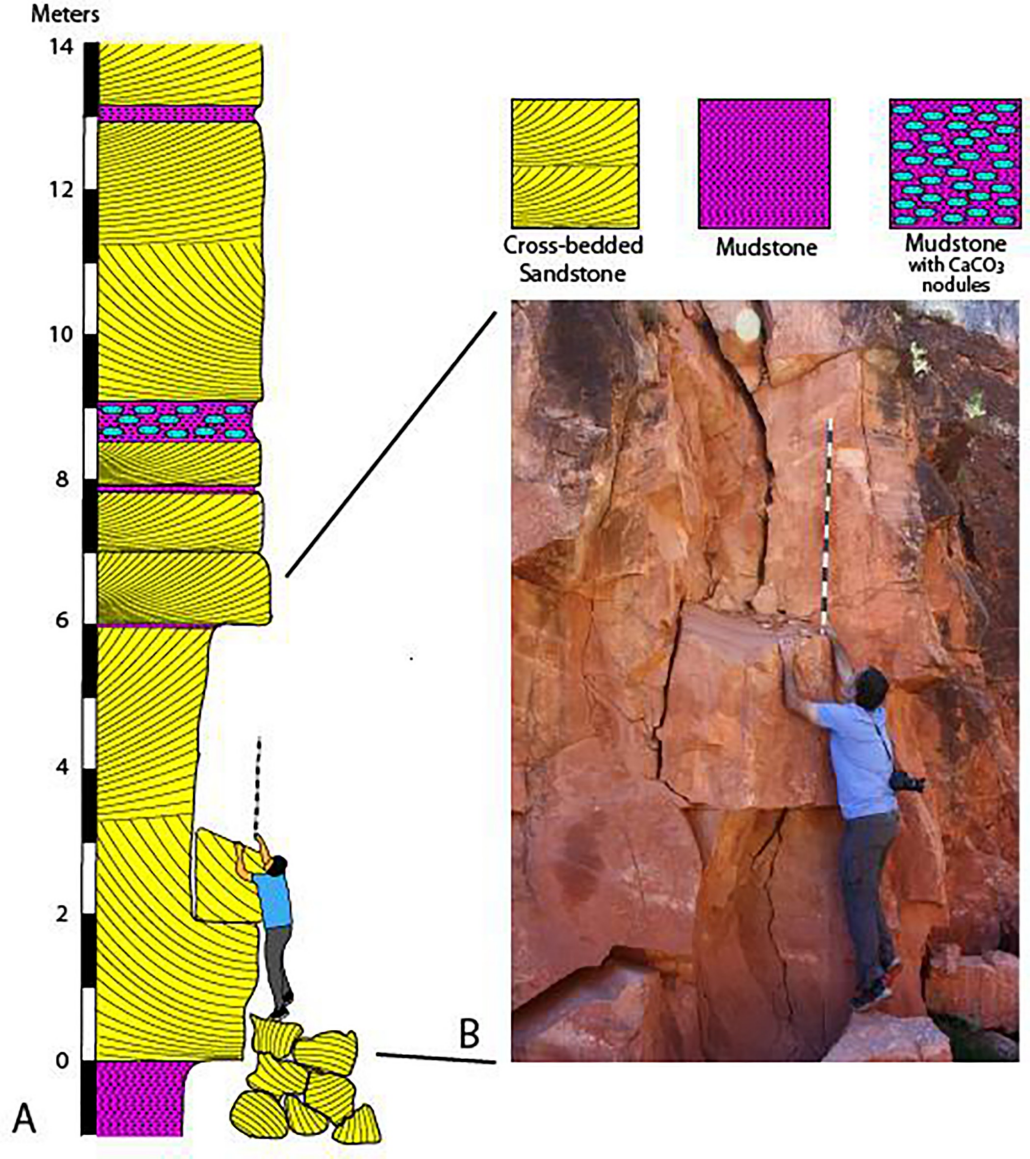
(*A*) Stratigraphic column/weathering profile and (*B*) photograph of cliff exposure of Manakacha Formation along the Bright Angel Trail. Cavity adjacent to calibrated staff is the presumed source of trackway-bearing rockfall blocks shown in Fig 2. Calibrated staff is 1.5 m.

The two uncollected, meter-scale blocks lie adjacent to a vertical cliff several meters high from which the rocks fell (Fig 3). There is no doubt that the source of the trackway blocks is this cliff face, and not a stratigraphic horizon higher up the slope; the lithology of the fallen blocks matches the lithology of the cliff face, and conspicuous voids in the cliff face record the source of rock falls from this cliff (Fig 3). This cliff face is well known to the National Park Service trail crew as a source of rock falls.

The locality occurs at an elevation of 1,539 m (5,050 feet) above sea level. Stratigraphically, the locality is about 30 m below the contact of the Manakacha Formation with the overlying Wescogame Formation [36]. The Manakacha Formation is 103 m thick along the Bright Angel Trail [37], so the trackway locality lies approximately 73 m above the base of the Manakacha.

Photogrammetry methodology involved taking overlapping photographs with a Canon Rebel XSi digital camera, perpendicular to the bedding plane from a distance of approximately 50 cm. Coded marker strips had first been taped to the bedding plane on both sides of Trackway 1. We used Agisoft Metashape Professional version 1.5.0 software to process the photographs and create a 3D model and false-color digital elevation model. Sedimentological analysis involved measurements of stratigraphic thickness with a calibrated staff, field identification of sedimentary structures, examination of rock textures and mineralogy with a hand lens, and examination of thin sections with a petrographic microscope.

### Ethical note

The person who appears in Fig 3B has provided written informed consent concerning his appearance in this photograph.

## Results and discussion

### Significance of the trackways

These are the first vertebrate trackways reported from the Manakacha Formation and the oldest known in the Grand Canyon region. They are among the oldest amniote trackways ever reported, and by far the oldest reported in eolianite. They provide serendipitous snapshots of two separate, possibly conspecific, animals employing very different gaits as they ascended a dunefield slope, thus capturing a record of ambulatory behavior in amniotes very early in this clade’s evolutionary history. The documentation of these trackways lowers the earliest-known occurrence of dunefield-dwelling amniotes to approximately 313 Ma, in the Moscovian/Atokan Stage (Westphalian in Western European biostratigraphic terminology) of the Pennsylvanian, very early in the history of amniotes (Fig 1). This age estimate is based on stratigraphic considerations discussed later in this paper.

We are in discussions with the National Park Service regarding the scientific importance of the Bright Angel Trail trackways, with the goal of placing both trackway-bearing blocks in a museum collection for protection from vandalism and weathering, possible public display, and additional research.

### Trackway 1

#### Description

**Fig 4:**
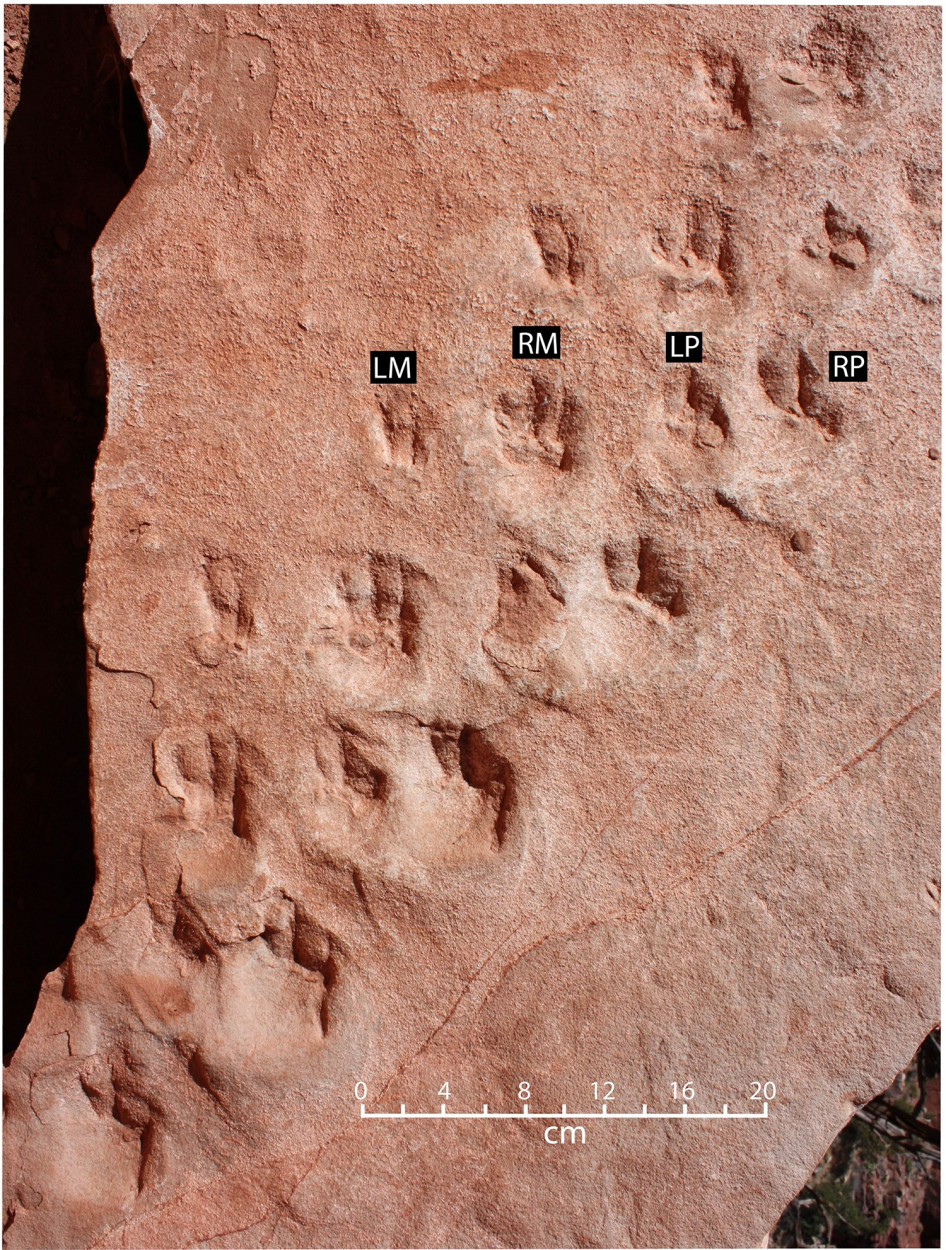
Photograph of a portion of Trackway 1. LM = left manus print; RM = right manus print; LP = left pes print; RP right pes print. Each footprint is interpreted to occur in the same position in each row, as indicated in the locomotion model (Fig 7).

**Fig 5:**
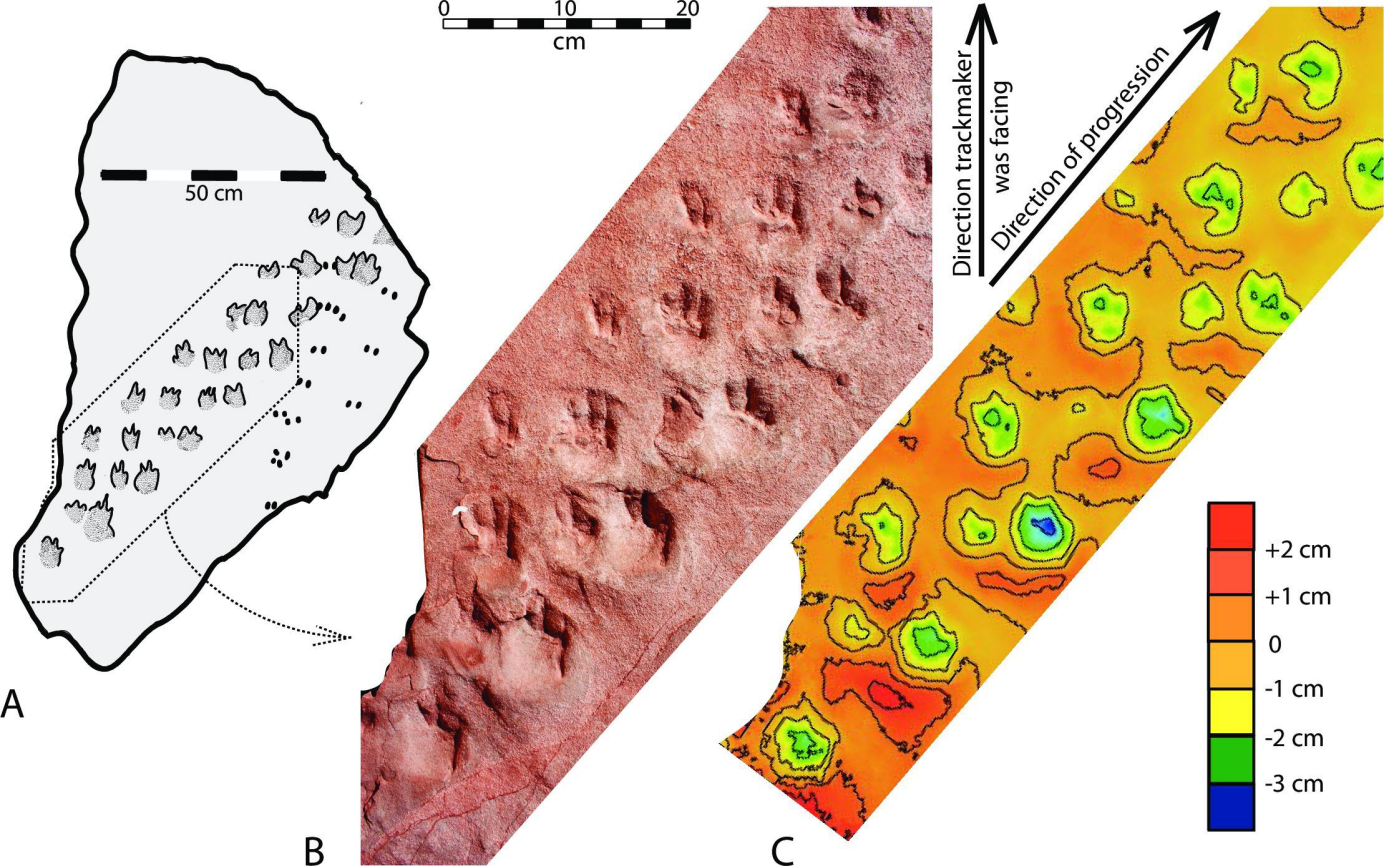
Trackway 1 and digital elevation model. (*A*) Sketch of main trackway surface. (*B*) Detail of a portion of the trackway, with scale. (*C*) Colored digital elevation model with explanation of colors. Contour interval is 1 cm. Scale is the same as (*B*).

Trackway 1 is 1.0 m long, consisting of twenty-eight tracks. The tracks are preserved in concave epirelief (impressions) on the main trackway block (Figs 2A and 4), and in convex hyporelief (natural casts) on the counterpart block (Fig 2D). This trackway consists of a series of parallel rows of tracks; each row consists of four tracks, and each row is offset to the right approximately 8 cm, in comparison with the previous row (Fig 4). Claw impressions in each footprint are pointed forward, but the direction of progression recorded in the trackway itself is oriented at a 40° angle to the direction that the animal was facing (Fig 5).

**Fig 6:**
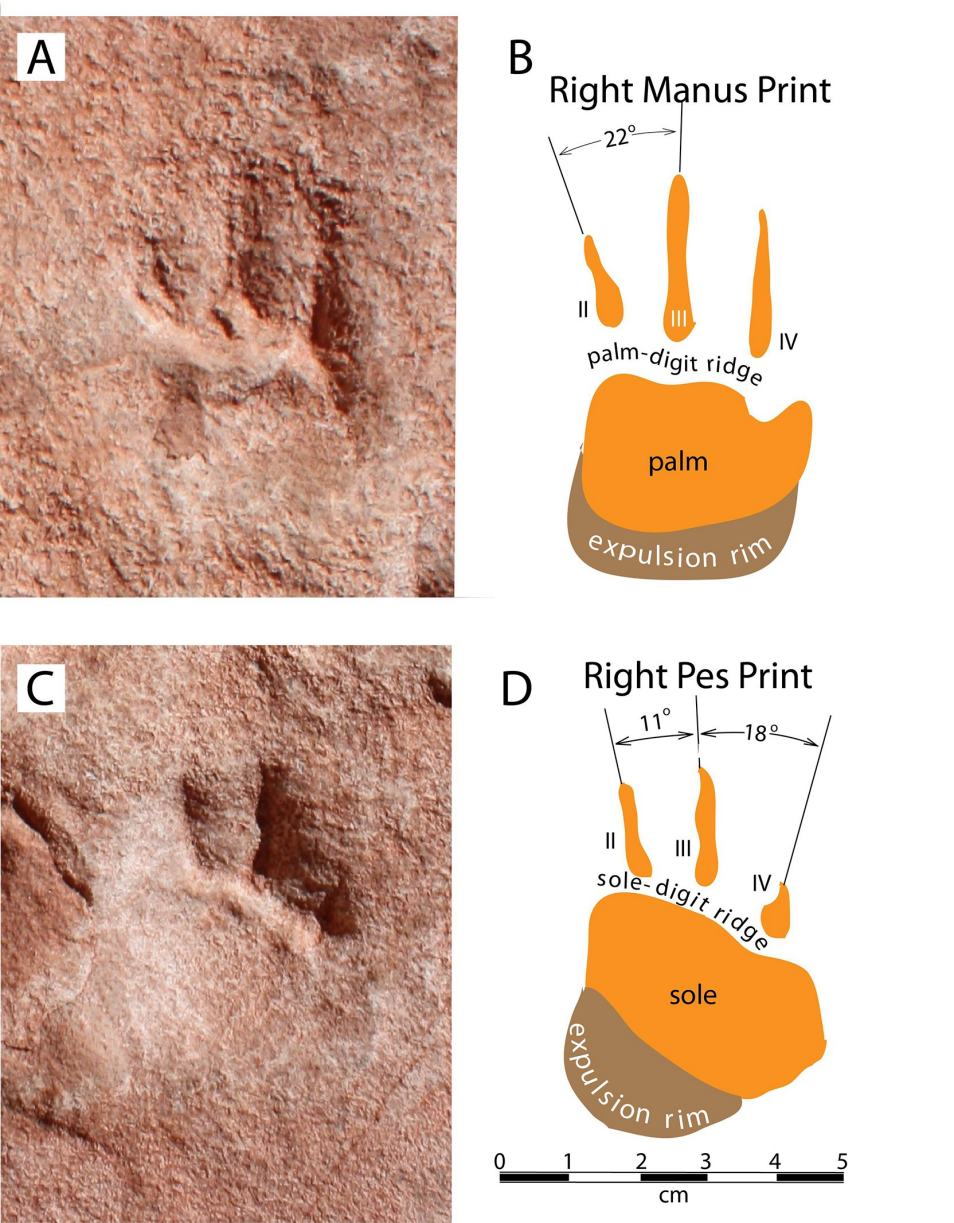
Detailed images of manus and pes prints. (*A*) Photograph of right manus print. (*B*) Sketch of right manus print. (*C*) Photograph of right pes print. (*D*) Sketch of right pes print.

The distinctions between right/left and manus/pes prints in Trackway 1, as labeled in Fig 4, are derived from our locomotion model, described below. Manus and pes prints are plantigrade, approximately the same size, with subcircular palm and sole impressions. Each is about 5 cm long and 3 cm wide with three straight digit traces terminating in sharp claw impressions (Fig 6). We infer that the three digit traces present in each print are those of digits II, III, and IV; digits I and V are presumed to have been present but were not impressed deeply enough into the substrate to register in these shallow undertracks. Digit traces are separated from the sole and palm by a prominent ridge. Digits on manus prints are longer than those on pes prints, with digit III being the longest in both cases. Digit IV pes grooves are much shorter than those of digits II and III. All three digits on both manus and pes prints are anteriorly directed, with small divarication angles (Fig 6).

#### Systematic Paleontology

These tracks are similar to the problematic ichnogenus *Chelichnus* Jardine 1850, as characterized by Haubold [38] and Citton et al. [39], although the manus prints of the Manakacha tracks are not conspicuously smaller than the pes prints, contrary to the typical pattern in *Chelichnus*. Compatible with the inference that the Manakacha trackmaker was pentadactyl, *Chelichnus* tracks typically consist of only three or four digits of a pentadactyl trackmaker [39]. Following a detailed examination of *Chelichnus* tracks in the Permian Coconino and De Chelly formations of Arizona, together with laboratory experiments with a live lizard, Marchetti et al. [34] considered *Chelichnus* to be *nomen dubium*, and they discarded it for ichnotaxonomic purposes. Tracks previously assigned to *Chelichnus* they assigned to other ichnogenera, mostly to *Varanopus*. However, we consider *Varanopus* to not be a suitable ichnotaxonomic assignment for the tracks in our Trackway 1. These tracks lack the ectaxonic digit morphology and very short palm/sole impressions characteristic of *Varanopus* [34]. Nor do the Manakacha tracks closely match other ichnogenera to which Marchetti et al. [34] referred other specimens formally known as *Chelichnus*. For the purposes of this paper, we refer to the Manakacha tracks as “*Chelichnus*-like,” with the expectation that additional discussion will occur within the ichnology community with regard to the ichnotaxonomic nomenclatural issues surrounding this ichnogenus, and also with the hope that additional specimens will turn up from the Manakacha Formation.

#### Taxonomic affinity of the Trackway 1 trackmaker

Impressions of three digits are present in each track (Figs 4 and 6), however no plausible Pennsylvanian candidate trackmaker taxon was tridactyl. Thus, we interpret the prints to be shallow undertracks made by a pentadactyl animal whose lateral digits were not impressed deeply enough into the sediment to translate into the preserved bedding plane. Without impressions of all five digits on each foot we are unable to measure foot slenderness and other characters that are useful for distinguishing among the tracks of various basal amniote taxa [7].

Because this trackway records the presence of relatively long digits with acuminate claws (Fig 6), we infer that the trackmaker was an amniote. As discussed below, this taxonomic inference also applies to the maker of Trackway 2. In the Pennsylvanian, the manus and pedes of non-amniote tetrapods were short and broad, with stubby digits [7,40], which clearly does not match these tracks. However, beyond the identification of the animal as an amniote, a confident taxonomic assignment is not possible. No single character can reliably be used to distinguish between the tracks of reptiles and those of basal synapsids (i.e., “pelycosaurs”) [41].

#### Locomotion model

**Fig 7:**
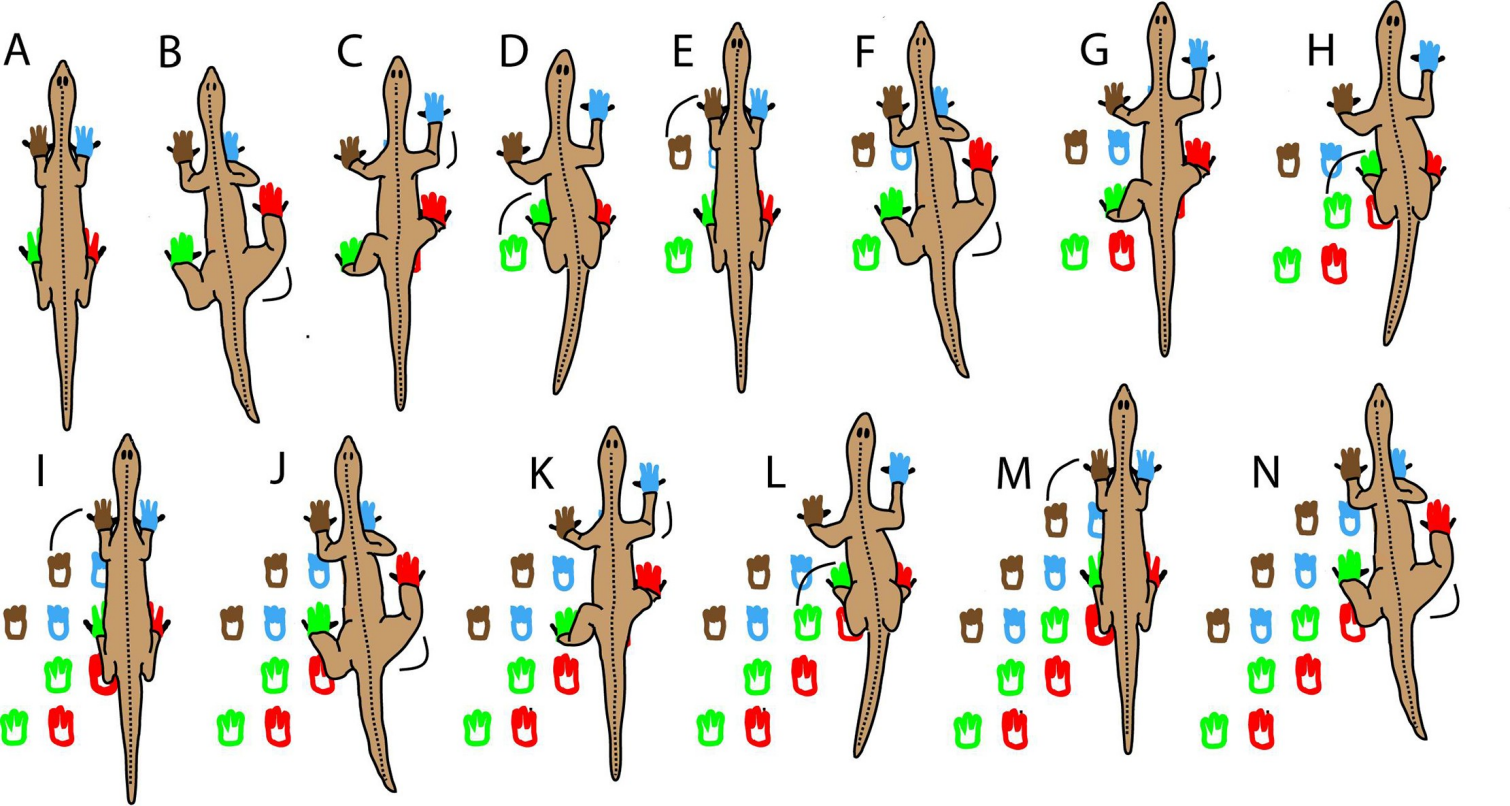
Lateral-sequence-walk locomotion model for creation of Trackway 1. Red outlines represent right pes prints; green outlines represent left pes prints; blue outlines represent right manus prints; brown outlines represent left manus prints.

At first glance, Trackway 1 appears to possibly record the passage of two animals, either walking side-by-side or sequentially. We reject this interpretation, however, primarily because of the remarkable regularity of footprint placement (Fig 4). Our locomotion model (Fig 7) accounts for this array of footprints to have been made by a single trackmaker. In our view, this is a simpler and more plausible explanation than an interpretation that requires two animals to have marched together or sequentially in lock-step precision, with no apparent overprinting of later steps on earlier ones.

As illustrated in our locomotion model (Fig 7), we interpret the unusual arrangement of footprints within this trackway to record a rightward-drifting, lateral-sequence walk; movement of the hindlimb on the right side of the body was directly followed by the forelimb on the right side. This was then followed, in succession, by the hindlimb and forelimb on the left side. As opposed to a diagonal-sequence gait, in which limb advancement on one side of the animal alternates with limb advancement on the opposite side, a lateral-sequence gait is the most parsimonious footfall-sequence interpretation that is compatible with the pattern of tracks in this trackway. Tetrapods, in fact, routinely use a lateral-sequence gait when walking slowly; while one foot is off the ground, this gait provides a larger stable triangle than other footfall sequences [42]. Moreover, a lateral-sequence gait facilitates undulations of the spine, which lengthen the step [42].

As indicated by expulsion rims adjacent to many of the tracks (Figs 4,5B,5C and 6), interpreted to occur on the downhill side, the trackmaker’s body was oriented straight up the slope. Presumably to lessen the steepness of its ascent, the animal repeatedly stepped forward and to the right, resulting in a diagonal traverse across the sloping surface and the 40° difference between the direction of progression and the direction in which the feet were pointed.

Fossil trackways that record diagonal movement on the slope of a sand dune are common in the ichnology literature [e.g., 24,34,43–46]. Francischini et al. [47, Fig 5A] documented an occurrence within the Permian eolian Coconino Sandstone of Arizona in which the angle of progression of an *Ichniotherium* trackway―inferred to have been made by the diadectid reptiliomorph *Orobates* [48]―differs markedly from the angle that the feet were pointing, similar to the case documented here in the Manakacha Formation (Fig 5). However, none of such previously documented cases of a tetrapod moving diagonally across the face of a sand dune record such a regular pattern of impressions of all four feet, as does Trackway 1 described here, and none have been interpreted to record a lateral-sequence gait. Camels routinely use a lateral-sequence pacing gait, which is recorded in Miocene fossil trackways [49], but we are not aware of reports of pre-Miocene occurrences of lateral-sequence gait in the tetrapod fossil trackway record.

**Fig 8:**
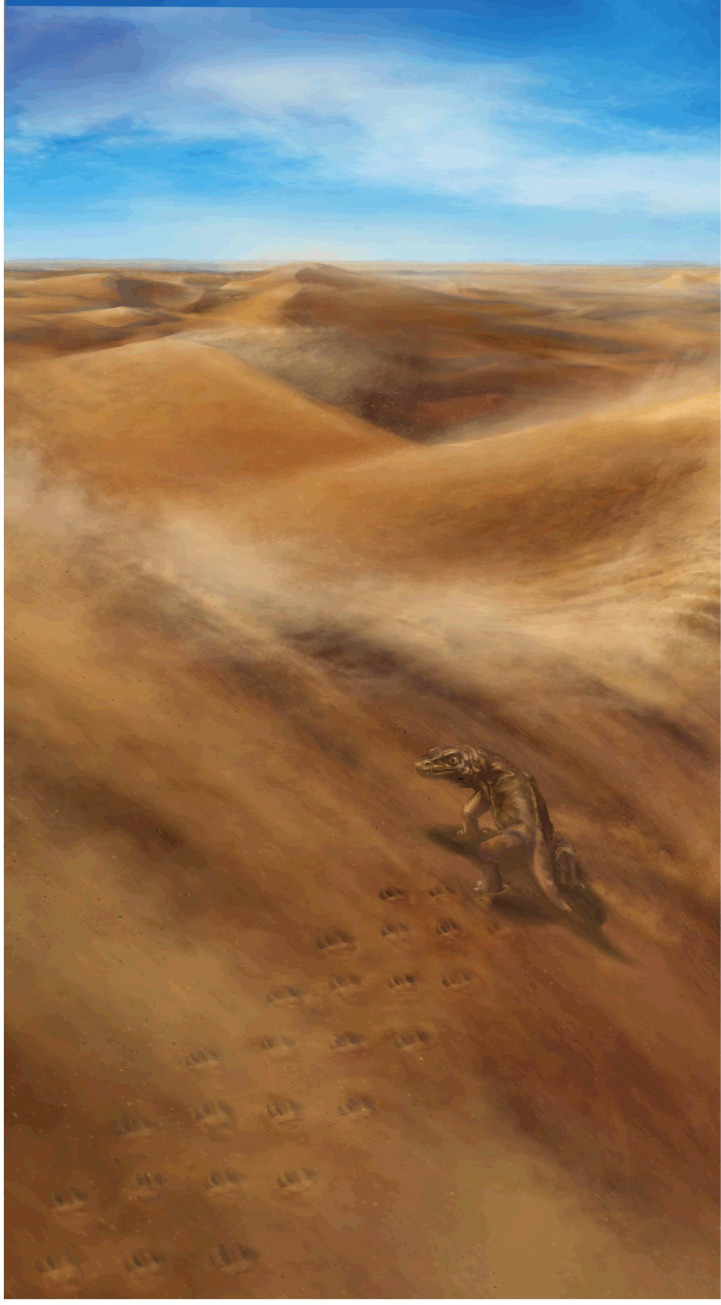
Artist’s rendering of a basal amniote moving diagonally up a sand dune, creating a trackway similar to Trackway 1 described in this paper. Art by Emily Waldman.

From a standing position with all four legs beneath it (Fig 7A), the animal thrust its right hind leg forward and to the right (Fig 7B). The footprint at the right end of each row of tracks (red in Fig 7)―interpreted in our model to be the print of the right pes―is consistently the deepest and most distinct (Figs 4,5B and 5C). These right-end prints also have conspicuous expulsion rims behind them, offset to the left, recording the rightward-veering, hindlimb thrust by the right hindleg. These features support our interpretation that the forceful extension and emplacement of the right pes was the lead motion within the four-step locomotion cycle. The right pes thus served as the anchor point for the remainder of the cycle; its emplacement was followed by the right foreleg being extended forward and to the right (Fig 7C). The prints of the right manus (blue in Fig 7)―second from the left in each row―are the second deepest prints, and they also have expulsion rims below them (Figs 4,5B and 5C). The left two legs then followed in succession, first the left hindleg (Fig 7D) and then the left foreleg (Fig 7E), in each case moving forward and to the right. The prints of these left feet (green and brown, respectively, in Fig 7) are shallower and without conspicuous expulsion rims. This rightward-drifting, lateral-sequence gait then continued, creating the pattern of tracks preserved in this trackway (Fig 7F–7N) and illustrated artistically in Fig 8.

The spacing of the footprints on this trackway is such that there was almost no overprinting of pes tracks on manus tracks. There is no consistent difference in the distance between the two manus tracks and the distance between the two pes tracks; both distances vary from 6 to 8 cm. If the trackmaker had been walking in a straight line, its stride would have been about 8 cm. The animal’s trunk length—the gleno-acetabular length—is taken to be the distance between the midpoint of the prints of the manus and those of the pedes at the beginning of a step cycle; that distance is about 18 cm (Fig 4). Following McKeever [44], we estimate that the animal’s total length was approximately double the trunk length, or about 36 cm.

#### Implications for the posture of Trackmaker 1

If our locomotion model is correct, the arrangement and spacing of Trackmaker 1 footprints (Fig 4) permits us to make inferences concerning the animal’s posture. The canonical paradigm of postural evolution in tetrapods consists of three stages: ‘sprawling,’ ‘semi-erect,’ and ‘erect’ [50]. Charig [51] modified this paradigm into an overtly progressivist sequence with the terms ‘sprawlers,’ ‘semi-improved,’ and ‘fully improved.’ Basal tetrapods were inferred to have had a sprawling locomotor posture, with their limbs extending laterally to the body, as occurs in salamanders and lizards [50,51]. According to the paradigm, this ‘primitive’ sprawling posture, which was inherited from rhipidistian fish [52], evolved into a semi-erect posture in some Paleozoic reptiles and synapsids. The semi-erect posture in turn evolved into the erect posture used by mammals and archosaurs; this latter development apparently coincided with the Permo-Triassic transition [53].

However, this unidirectional, progressivist paradigm of evolving postural grades has been challenged as being over-simplified, and it blurs important differences between members of each grade [54,55]. The notion that the transition from one grade to the next represents an improvement in locomotor efficiency was dispelled by the demonstration that ‘sprawling’ is not more expensive energetically than moving with an ‘erect’ posture [54,56]. Crocodilian locomotion also challenges the paradigm. Modern crocodilians use both a sprawling and a semi-erect posture, so they were commonly thought to represent an example of the sprawling-to-erect transition. However, Reilly and Elias [55] showed evidence that the locomotor postures of crocodilians were secondarily evolved from erect ancestors.

It cannot be assumed *a priori*, therefore, that basal amniotes such as the Manakacha trackmakers utilized a sprawling posture. In recent studies, femoral abduction angle―the smallest angle between the sagittal plane and the longitudinal axis of the femur―among other measurements, has been used to quantitatively characterize limb posture [e.g., 53,57]. While we cannot directly measure the femoral abduction angle of the trackmaker, the tracks provide direct evidence of locomotory posture. In the case of Trackmaker 1 in our study, each step cycle began with the manus and pedes close together (Fig 7), which implies a relatively small femoral abduction angle and a relatively erect posture. We explore the question of limb posture in more detail below, in our interpretation of Trackway 2.

### Trackway 2 description and interpretation

**Fig 9:**
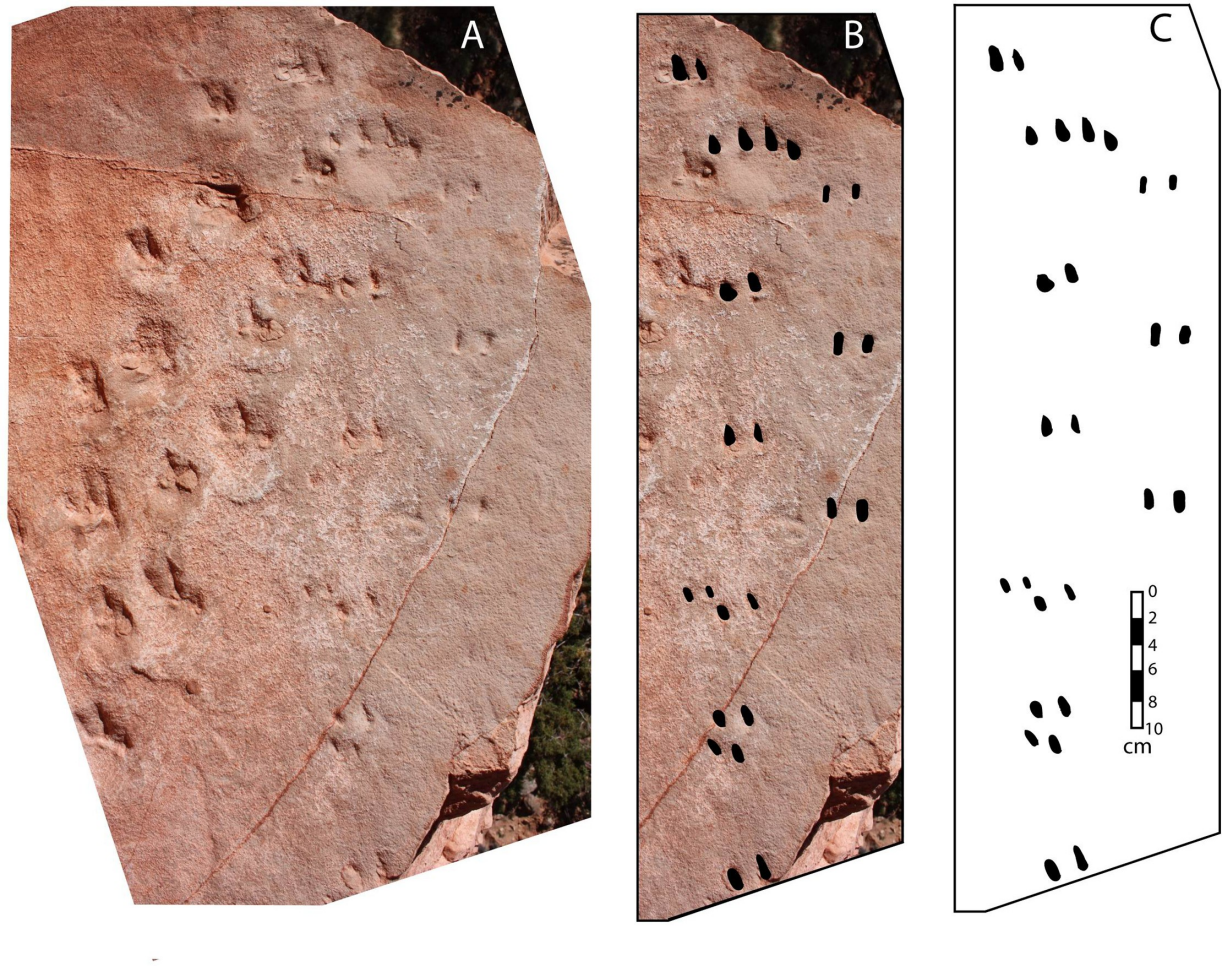
Trackway 2. (*A*) Photograph showing Trackway 1 (left) and Trackway 2 (right). Note that Trackway 2 is parallel to direction in which tracks in Trackway 1 are pointing. (*B*) Photograph showing Trackway 2, with pits blackened for emphasis. (*C*) Sketch showing Trackway 2. Scale is the same in all three images.

In addition to Trackway 1, discussed above, the same bedding plane contains two parallel alignments of pairs of oval-to-teardrop-shaped depressions (Fig 9). The individual pits range in length from 3 to 8 mm; members of the same pair are about 2 cm apart. We interpret these two parallel sets of depressions to be a deeper undertrackway, Trackway 2, that is separate from―and slightly younger than―Trackway 1. According to this interpretation, additional sand had accumulated above the Trackway 1 surface, perhaps a centimeter or more in depth, and an animal with acuminate claws walked up the same slope while the sand in which the earlier-formed trackway had been impressed was moist. Two of the animal’s claws on three of its feet apparently penetrated just deeply enough to leave small impressions preserved in the same bedding plane in which Trackway 1 had been preserved, perhaps a few hours or days before. The pits that are teardrop shaped consistently taper in the same direction that the earlier trackmaker was headed, from which we infer that Trackmaker 2 was also walking *up* the slope. The left set of pits (Fig 9C) preserves claw marks of the left manus and left pes, while the right set of pits preserves the claw marks of just one of the feet. One set of pits on the right side, as well as some of the claw marks on the left side, are ‘missing.’ The ‘missing’ row of claw marks indicates that one of the right feet, and occasionally one of the left feet, was not being implanted deeply enough to penetrate down to the preserved bedding plane, possibly due to an injured right leg.

**Fig 10:**
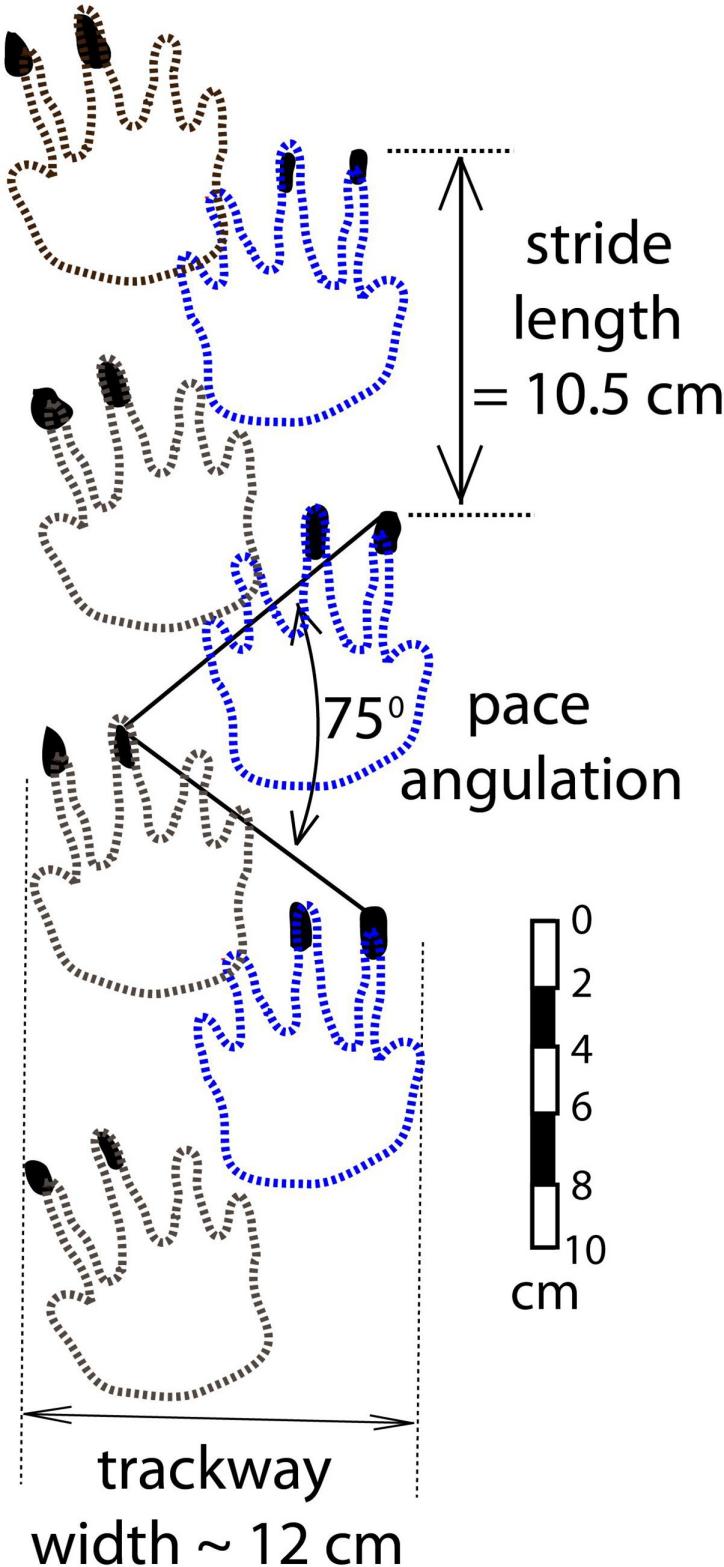
Reconstruction of Trackway 2 using the most persistent and regularly spaced claw marks, together with outlines of pentadactyl feet based on the manus prints of Trackway 1.

Our interpretation of Trackway 1 indicates that the manus claws of the trackmaker were longer than its pes claws (Fig 6). Following the working hypothesis that the two trackmakers were conspecific, we postulate that the most persistent and regularly spaced sets of paired claw marks in Trackway 2 are manus impressions. We further postulate that the paired claw marks represent digits III and IV, the longest of the three manus digit traces in Trackway 1 (Fig 6A and 6B). In Fig 10 we have superimposed outlines of pentadactyl footprints, generalized from the Trackway 1 manus prints (Fig 6B), onto the most persistent, regularly spaced sets of paired claw impressions of Trackway 2 (from Fig 9C). This provides an opportunity to measure parameters of reconstructed Trackway 2: the stride length is 10.5 cm, the pace angulation is 75°, and the trackway width is about 12 cm (Fig 10). This trackway width is somewhat wider than that of Trackway 1 (measured as if the animal had been walking in a straight line), but it is of course not possible to compare them precisely; Trackway 1 tracks are missing digits I and V, and each of the Trackway 2 tracks consists of only two claw marks. Moreover, we infer that Trackway 1 was made by an animal using a lateral-sequence gait, moving diagonally across a slope, while the arrangement of tracks in Trackway 2 indicates that it was made by an animal using a diagonal-sequence gait, while it walked straight up the slope. Such a difference in gaits could easily account for differences in trackway width. Based on our analyses of the morphology of both trackways, we conclude that Trackmaker 1 and Trackmaker 2 could have been made by animals belonging to the same species, or conceivably even the same individual.

Trackmaker 2 was evidently ascending the slope nearly in a straight line and more rapidly than was the side-stepping Trackmaker 1. Whether or not these two trackmakers were conspecific, for reasons discussed above with respect to the taxonomic affinities of Trackmaker 1, a Pennsylvanian tetrapod with clawed digits, such as Trackmaker 2, must also have been an amniote.

#### Limp, posture, and estimated speed of Trackmaker 2

The reconstructed manus tracks of Trackway 2 reveal a slight limping gait in which the distance between successive right and left prints within each step cycle is consistently slightly less than the distance between successive left and right prints (Fig 10). This slight limp reinforces the suggestion that one of this animal’s right legs was compromised.

Because of the short stride, the reconstructed trackway displays a very low stride-length to trackway-width ratio of 0.9. Such a ratio is typically characteristic of a tetapod with a low hip height and a sprawling gait [44]. However, because the animal was ascending a slope, its stride length was shorter than it would have been, had it been walking on level ground. The reconstruction (Fig 10) indicates that the medial margins of the right and left footprints overlapped one another, which would not occur if the animal had a sprawling posture.

A recent biomechanical study of the Lower Permian diadectid reptiliomorth *Orobates* revealed that ‘advanced’ terrestrial locomotor properties, such as a relatively erect posture, were present in this animal. The authors concluded that such characteristics were also present in the last common ancestor of diadectomorphs and amniotes, which was ancestral to the Manakacha trackmakers (Fig 1). Pace angulation, measured in trackways, has been used as a proxy for limb posture [57]. Pace angulation in the Permian ichnogenus *Ichniotherium* ranges from 70° to 110° [47]. The 75° pace angulation of Trackmaker 2 in our study also falls within this range (Fig 10). As with the short stride, we attribute the low pace angulation to the fact that the animal was ascending a slope.

Based on the narrowness of Trackway 2, we infer that this animal had a relatively small femoral abduction angle and a relatively erect posture. As discussed above, our locomotion model based on Trackway 1 (Fig 7) implies a relatively erect posture for that trackmaker also. We conclude, therefore, that the postures of both trackmakers were surprisingly erect for basal amniotes.

With appropriate caution, we can use the stride length and foot length to estimate the speed of Trackmaker 2. Alexander [58] studied locomotion in living terrestrial vertebrates and obtained the following relationship between speed *u*, hip height *h*, and stride length λ:

$$\lambda /h=2.3{\left(u^{2}/gh\right)}^{0.3}$$

where *g* is acceleration due to gravity. McKeever [44] applied this formula to several ichnospecies of tetrapods in the Permian of Scotland, including *Chelichnus* ispp. We adopt his approach with our analysis of Trackway 2. The challenge, of course, is to determine the hip height of the trackmaker. If we adopted McKeever’s [44] working approximation for Late Paleozoic tetrapods that the hip height was 1.5 times the pes length (which assumes a sprawling posture), using a pes length of 5 cm (Fig 6D) and the Alexander formula cited above, Trackmaker 2 would have been walking at a speed of 0.38 m/sec or 1.37 km/hr. However, because the close distance in our reconstruction between the right and left tracks (Fig 10) implies a relatively erect posture, our preferred multiplier for estimating the hip height is four times the pes length [58]. Using that value yields a slow, plodding speed of 0.12 m/sec or 0.43 km/hr. This speed is considerably slower than any of the rates calculated by McKeever (44) for several Permian trackways in Scotland referred to the ichnogenus *Chelichnus*. These differences are largely due to the fact that McKeever assumed a low hip height.

For comparison of our calculated Trackmaker 2 speed with the speed of an extant species of lizard of about the same size, on flat ground the Galapagos marine iguana, *Amblyrhynchus cristatus*, can maintain a walking speed of about 1 km/hr for at least 20 minutes, and it is capable of brief sprints at a speed of 9 km/hr; in spite of its adaptation to swimming, this lizard is agile on dry land [59]. Our data suggest that Trackmaker 2 ascended a 20° slope at a rate of about 0.4 km/hr, a speed that would be unremarkable for a modern lizard of comparable size. Like the marine iguana, Trackmaker 2 could probably exhibit more nimble behavior under different circumstances.

### How were the trackways preserved?

Dune sand may seem to be an unlikely medium in which to preserve distinct footprints and undertracks. However, under optimum circumstances such preservation has been shown to occur [24,32,33,60–64]. Experiments have shown that clearly defined tracks of lizard-size animals in eolian sand are made only when the sand is dry [34,60]. This includes tracks made by large lizards such as chuckwallas (*Sauramalus* sp.), which are comparable in size to the Manakacha trackmakers [60]. However, permanent preservation of such tracks, as well as the development of a parting surface along which the tracks can be later exposed, requires the surface to become dampened [60]. Even after the dampened surface dries, a crust of cohesive grains remains. If dry sand later covers this cohesive crust, the interface between the crust and the overlying interval of dry sand may be preserved in the rock record as a horizon of weakness along which the rock may split open [60].

In the case of the two Manakacha Formation trackways described here, we postulate the following sequence of events: (1) Trackmaker 1 diagonally ascended a gentle slope of eolian, dry, quartz sand, employing a lateral-sequence gait, leaving a distinct trackway; (2) the uppermost few millimeters of the trackway surface were subsequently eroded, due to high wind speed and a paucity of loose sand, removing the prints of digits I and V, and leaving Trackway 1 as the shallow undertrackway that is exposed today; (3) the exposed undertrackway was then dampened by fog, dew, or light rain, creating a surficial crust of moist, compact, sand grains; (4) dry sand then covered this moist crust, perhaps one or two cm deep; (5) Trackmaker 2 then happened along, ascending straight up the same slope, moving more quickly than Trackmaker 1 had moved, employing a diagonal-sequence gait, digging its claws deeply enough for some of them to penetrate the buried but still damp Trackway 1 horizon, thereby creating Trackway 2; (6) the sand was subsequently buried, compacted, and cemented with calcite; (7) following uplift, exposure due to down-cutting by the Colorado River, and weathering, a boulder-size rock fell from a cliff along the Bright Angel Trail, and it split open along a weak parting surface that had been created approximately 313 million years earlier.

### Extension of the *Chelichnus* ichnofacies into the mid-Pennsylvanian

*Chelichnus*, in spite of its dubious status as an ichnogenus, is the eponymous ichnotaxon of the *Chelichnus* ichnofacies. This ichnofacies is a recurring, low-diversity assemblage of tetrapod tracks in eolian lithofacies that is known from the Early Permian to the Early Jurassic [65]. *Chelichnus* also figures prominently in a recently proposed episodic model of Phanerozoic colonization of deserts; Krapovickas et al. [66] used *Chelichnus* and the *Chelichnus* ichnofacies to characterize the third phase (Carboniferous-Permian) of their five-phase, desert-colonization model, although *Chelichnus* itself had not been known prior to the Early Permian. The documentation of *Chelichnus*-like tracks in the mid-Pennsylvanian Manakacha Formation now fills the gap between the dawn of desert colonization by tetrapods in the Pennsylvanian and the previously-known oldest occurrence of the *Chelichnus* ichnofacies in the Early Permian.

### Stratigraphy

The age of the Manakacha Formation is tightly constrained to the Atokan North American Stage by the presence of a well-documented fauna in marine facies [67,68] (Fig 1). The Atokan Stage ranges from about 319 Ma to about 312 Ma [69]. Moreover, the Watahomigi Formation, which unconformably underlies the Manakacha, also contains Atokan marine fossils in its youngest strata [67]. These stratigraphic and biostratigraphic relationships thus indicate that the Manakacha occupies the upper two thirds of the Atokan Stage, ranging in age from approximately 316 Ma to approximately 312 Ma (Fig 1). The Bright Angel Trail trackways occur about three quarters of the distance up from the base to the top of the Manakacha. Allowing for a modicum of variation in sedimentation rate, we estimate the age of the tracks to be 313 ± 0.5 Ma (Fig 1).

### Sedimentology

**Fig 11:**
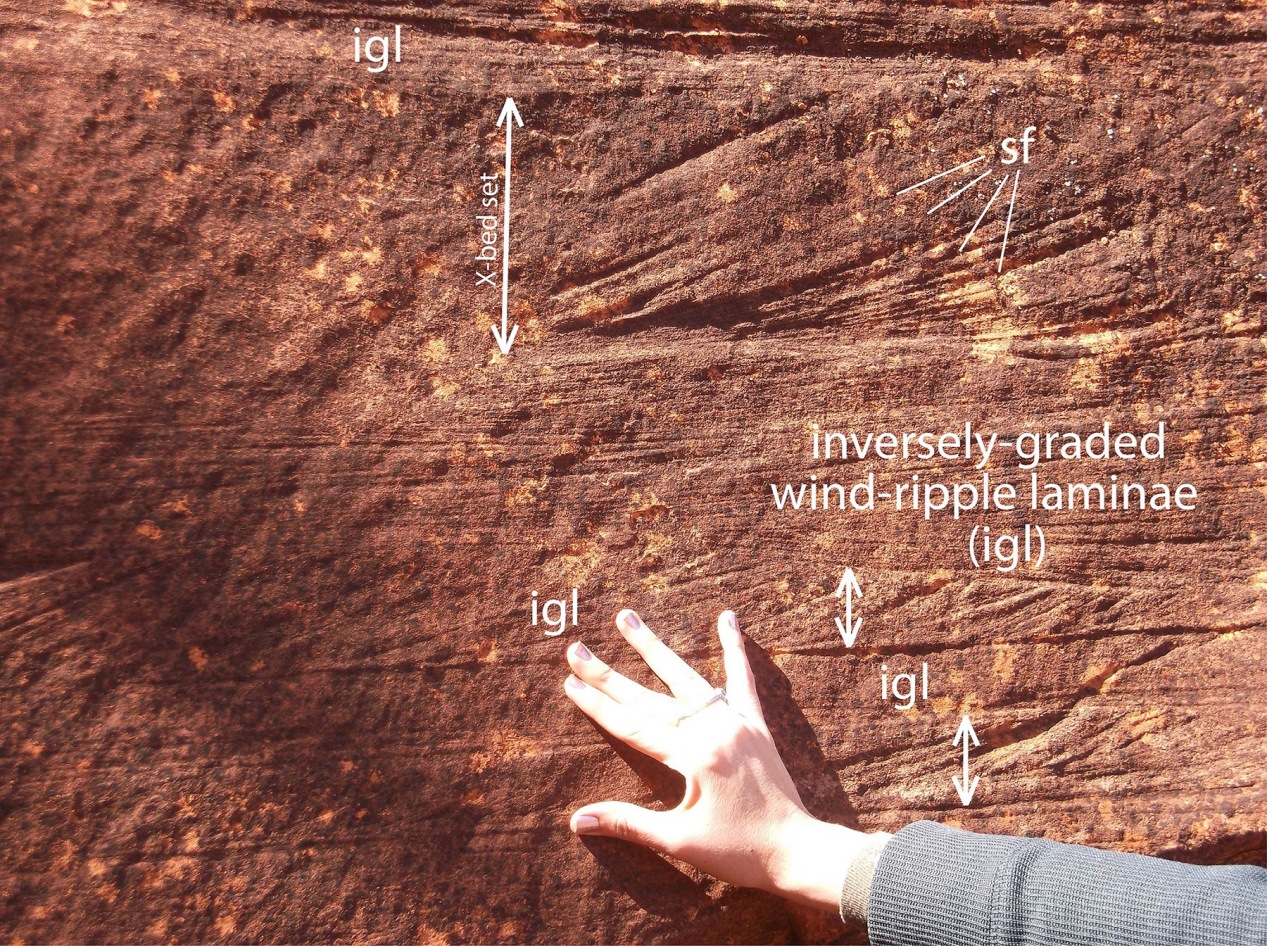
Exposure of eolian sandstone bed of the Manakacha Formation on the Bright Angel Trail near the tracksite. Internal structure: (1) cross-bed sets (double-pointed arrows) composed of sparse, thin sandflows (sf; labeled in upper example) that wedge-out down foreset dip into tangential toesets of wind-ripple laminae, and (2) interbeds of inversely-graded wind-ripple laminae (igl).

**Fig 12:**
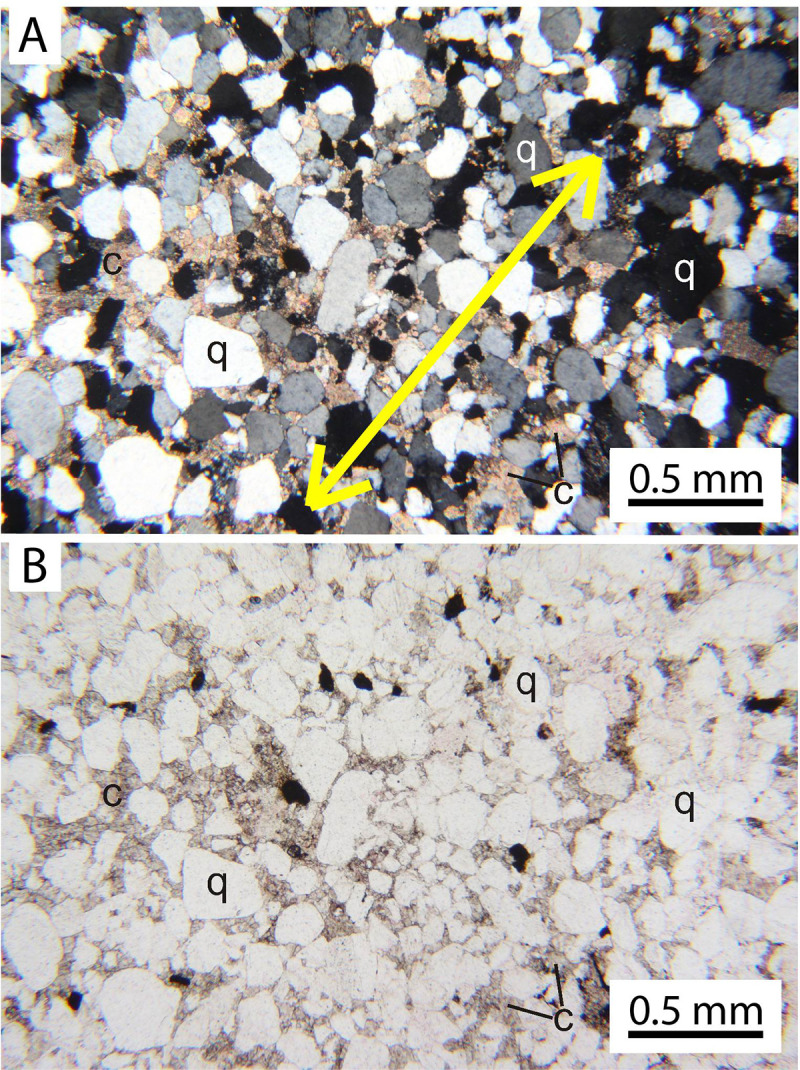
Photomicrograph of a Manakacha Formation sandstone thin section from the main trackway block. It is quartzarenite with rounded, medium- and fine-grained quartz grains (q), ≤5% lithic fragments, cemented with calcite (c). (*A*) With nicols crossed. (*B*) In unpolarized light. Small dark grains in (*B*) are opaque oxides, such as ilmenite and magnetite. The sample from which this thin section was made had broken off the main trackway block by natural processes, so its orientation with respect to bedding is not known. Yellow arrow in (*A*) identifies a possible upward-coarsening wind-ripple lamina.

The Manakacha Formation is a mixture of eolian, fluvial, and marine lithofacies, with eolian sandstone being the dominant lithofacies in central Grand Canyon [67,70–72]. As exposed along the portion of the Bright Angel Trail where the trackway blocks occur, the Manakacha Formation consists of a succession of tan, cliff- and ledge-forming, cross-stratified sandstones interbedded with dark reddish-brown, recess-forming mudstones. Cross-bed sets range in thickness from a few cm to several m (Figs 3 and 11). They are composed of fine-to-medium-grained quartz sand in upward-coarsening laminae that average 2 mm in thickness (Figs 11 and 12). The few, localized sandflows are fine- to medium-grained beds up to 3 cm thick; they taper down-dip into toesets of upward-coarsening laminae in cross-bed sets (Fig 11). No grainfall strata were recognized in the Bright Angel Trail exposures. The main track-bearing block of sandstone is moderately- to well-sorted, fine- to-medium-grained quartzarenite with rounded grains (Fig 12).

The interbedded mudstones are structureless to thinly laminated; they range in thickness from 1 cm to 2 m (Fig 3). One mudstone bed contains calcareous nodules (Fig 3), recording a diagenetic phase that may have been contemporaneous with the precipitation of the calcite that cements the grains of the sandstone (Fig 12). Some of the mudstones display features indicative of soft-sediment loading and squeeze-up into the overlying cross-bedded sandstones.

We interpret the dominant upward-coarsening laminae in the track-bearing blocks to be wind-ripple strata. When present, such laminae are the most reliable indicator of eolian sedimentation [73–75]. Localized sandflows are the product of avalanches or grainflows on unstable eolian-dune lee slopes (Fig 11) [75]. In Bright-Angel-Trail exposures of the Manakacha Formation, sandflow laminae are sparsely developed relative to upward-coarsening, wind-ripple laminae; this may be related to a high degree of grain cohesion caused by rainfall, coastal moisture, or the fact that these deposits formed in a tropical latitude where the humidity was high.

In eolian settings, wind ripples typically occur on flats between dunes, at the bases of dunes, and up the faces of dunes. Within a prominent cavity in the cliff face (Fig 3), such laminae have a dip of about 20°. This cavity is the probable source of the trackway blocks, which leads us to infer that the trackmakers were ascending a slope of approximately 20°, or possibly steeper prior to compaction and flattening of the dipping laminae. The presence of prominent expulsion rims on the rear margins of tracks of Trackway 1 (Figs 4–6) reinforces the inference that the animals were ascending a gently sloping surface in the toe region of a dune, where relatively firm, tightly-compacted, wind-rippled sand had been deposited.

We interpret the depositional setting of the trackway-bearing interval and associated strata to have been a coastal-plain, eolian dunefield. The sandstone-mudstone cyclicity resulted from eolian dunes that were episodically interrupted by tidal or wadi flooding and buried by mud in a water-saturated state. Interacting eolian-subaqueous processes resulted in the sandstone-mudstone cycle, controlled by either sea-level rises and fluctuating marine shorelines or seasonal rainfall pulses that caused wadi flooding of the dunefield. Burrowing organisms disrupted laminations in most of the mudstones, rendering them structureless. Subsequent wind events caused eolian dunes to migrate across and load the soft mudstones.

**Fig 13:**
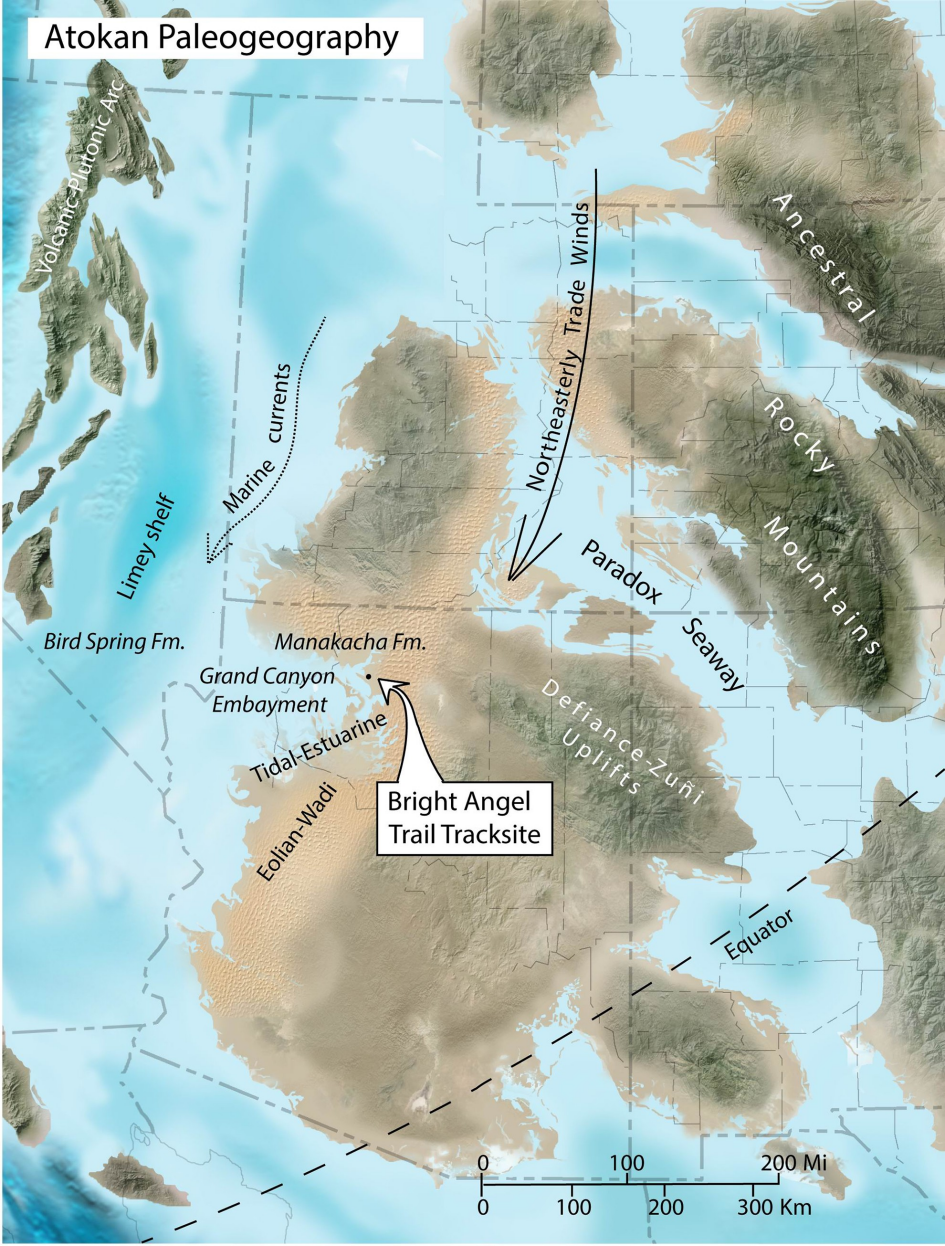
Paleogeography of southwestern North America during deposition of the Manakacha Formation. Background image is updated from [76,77]; copyright, 2012, Colorado Plateau Geosystems; courtesy of Ron Blakey; used with permission. All of the labels are ours. Present-day north is straight up. Position of the paleo-equator is from [26,81]. Dashed lines on background map are present-day state and county boundaries; Arizona occupies the central portion of the lower half of the figure, Utah occupies the central portion of the upper half.

The ‘Atokan Lowstand,’ during which the Manakacha Formation was deposited, corresponds to a cyclothemic interval in the Midcontinent, and it coincides with a major glaciation in Argentina and Australia [30]. Correlative, carbonate-dominated, marine deposits in the southern Great Basin are those of the Bird Spring Formation (Fig 13). Sequence stratigraphic analysis of the Atokan portion of the Bird Spring Formation has revealed a pattern of rhythmic cycling of inner-platform and restricted platform-interior facies assemblages arranged in three stratigraphic sequences, all roughly equal in thickness [30]. These rhythmic miogeoclinal cycles record a relatively constant rate of subsidence and sedimentation on the margin of Atokan Euramerica, where the marine strata of the Bird Spring Formation and the correlative landward, non-marine strata of the Manakacha Formation were deposited.

### Paleogeography

Paleogeographic reconstructions of southwestern North America in the Atokan Age, show the study site to have lain near the western margin of a large, low-latitude island that encompassed most of what is now Arizona and portions of adjacent states [76,77] (Fig 13). Proterozoic crystalline rocks and lower Paleozoic strata had been uplifted and exposed to the east, forming the Ancestral Rocky Mountains. These were separated from the Defiance-Zuñi Uplifts by the Paradox Seaway, which lay on the eastern margin of this Arizona-size Atokan island (Fig 13). Coarse, siliciclastic sediments from the Defiance-Zuñi Uplifts were transported westward across the island, including eolian sand and silt driven by northeasterly trade winds [26,76]. Pulses of sea-level rise episodically flooded this region, interleaving carbonates and evaporites with the non-marine strata [71,77–80].

The Manakacha Formation is a product of this paleogeography. The eolian sandstones of the Manakacha Formation represent the earliest record of a paleowind system in the American Southwest [67,70,71]. They mark the initiation of a protracted interval of dominantly eolian sedimentation that continued for more than 100 million years, into the Jurassic Period, providing habitats for countless dunefield-dwelling organisms.

The westward-marching Manakacha eolian dunes eventually foundered in the tidal-estuarine settings of Grand Canyon Embayment (Fig 13) [77]. Farther westward, on a limey, nearshore shelf, carbonate sediments on exposed shoals were reworked into cross-stratified carbonate eolianites [72]. The North American portion of Pangaea in the Carboniferous was rotated approximately 45° clockwise, relative to its present-day orientation, and the supercontinent was drifting northward [14]. The Colorado Plateau region lay roughly 20° south of the equator in the mid-Mississippian, at about 340 Ma; thirty million years later, at 310 Ma, in mid-Pennsylvanian time, the Colorado Plateau region lay a few degrees north of the equator [14]. Our placement of the paleo-equator in Fig 13, which places the study area at a latitude of about 4°N, represents a reconstructed position at about 300 Ma, at the end of the Carboniferous [26,81]. Thirteen million years earlier, when the Bright Angel Trail tracks were made, the tracksite was probably even closer to the equator than shown in Fig 13. This is a surprisingly equatorial position for an arid dunefield. A modern example of low-latitude dunes is the occurrence of dormant and relict dunes at a latitude of approximately 5°S, in the northern Kalahari Desert of West Africa [82], but such low-latitude dunes are not common. We suggest that the Ancestral Rocky Mountains and Defiance-Zuñi Uplifts were partial barriers to westward air flow in the Trade Wind Belt, creating the Manakacha rain-shadow desert (Fig 13).

U-Pb ages of detrital zircons in the Manakacha indicate that sand grains in the Manakacha Formation were derived from two sources: (1) the Ancestral Rocky Mountains (and/or other regional sources, such as the Defiance-Zuñi Uplifts), and (2) the Appalachian Orogen [83]. Appalachian grains were presumably transported westward from eastern North America in continent-wide fluvial systems and deposited into the sea, north of the Colorado Plateau region; from there they were transported southward by marine currents and then reworked into eolianites of the Manakacha and younger formations [84].

### Regional stratigraphic and ichnological context

**Fig 14:**
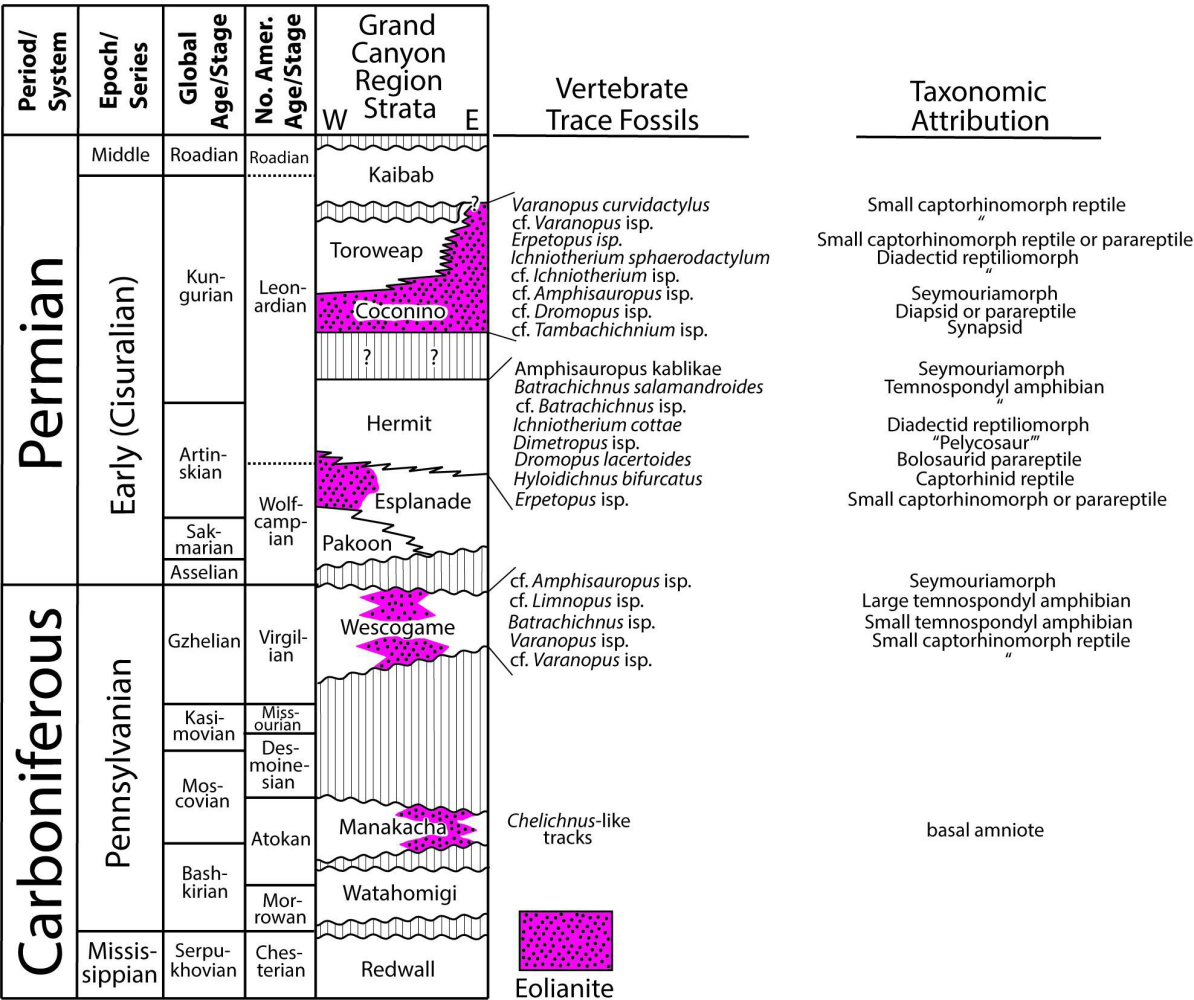
Time-rock stratigraphic diagram of Pennsylvanian and Permian strata of the Grand Canyon region, showing the occurrence of eolian sediments and vertebrate trace fossils. Diagram is adapted from [67], with the occurrence of eolianite adapted from [70]. Occurrences of vertebrate trace fossils are from [86] and this paper. Vertical axis is not precisely to scale.

To place the Manakacha trackways described here in a regional stratigraphic and ichnological context, in Fig 14 we summarize the Pennsylvanian-through-Permian stratigraphy of the Grand Canyon region and the occurrences within these strata of vertebrate trace fossils. The southern Colorado Plateau/Grand Canyon region is the best-exposed, best-studied depocenter on Earth for Pennsylvanian and Permian trace-fossil-rich eolianites. Until now, the earliest known occurrence of tetrapod tracks in the Grand Canyon region was a low-diversity ichnofauna in the latest Pennsylvanian (Gzhelian/Virgilian) Wescogame Formation (Fig 14). This formation is roughly correlative with the Weber Sandstone, discussed above, or is possibly slightly older. The Wescogame ichnofauna was originally described by Gilmore [85]. It was revised by Santucci et al. [45] and later by Marchetti et al. [86]. The Wescogame consists predominantly of marine shelf and shoreline siliciclastic and carbonate facies, with some eolian and some fluvial strata [67,70,72], however the specific depositional environment of the track-bearing strata within the Wescogame has not yet been studied. We are not aware of well-dated pre-Gzhelian/Virgilian occurrences of vertebrate-trace-fossil-bearing eolianite elsewhere in the world, other than those described in this paper.

In addition to the tracks in the Wescogame Formation, vertebrate tracks have also been reported from the Hermit Formation, and Coconino Sandstone [24,27,45,68,85–94] (Fig 14). The Hermit is predominantly fluvial, while the Coconino is exclusively eolian [67,70]. The ichnotaxonomy of the Coconino ichnofauna has recently undergone a major revision [34,86].

## Conclusions

Two trackways are preserved on part-and-counterpart, bedding-plane surfaces within eolian sandstones of the Manakacha Formation in Grand Canyon National Park. They record the occurrence of two, possibly conspecific, amniote trackmakers with small femoral abduction angles and correspondingly erect postures. These animals ascended a slope of approximately 20° in an Atokan eolian dunefield 313 ± 0.5 million years ago, very early in the history of amniotes. Trackmaker 1, whose tracks are *Chelichnus*-like, employed a right-drifting, lateral-sequence gait as it ascended diagonally across the slope. This is the first documented occurrence of a lateral-sequence gait in the pre-Miocene, tetrapod fossil record. Trackmaker 2 came along sometime later, after more sand had accumulated on the surface. It ascended more quickly than Trackmaker 1, but it was still moving slowly, at a rate of approximately 0.1 m/sec (~0.4 km/hr), going straight up the slope using a diagonal-sequence gait. These trackmakers were living in an eolian-dunefield desert setting on a low-relief, equatorial, coastal plain upon which tidal or wadi flooding episodically interrupted eolian processes. These trackways document the adaptation by amniotes to eolian sand dunes very soon after this clade evolved, at least eight million years earlier than previously documented.
